# Bilateral Grynfeltt lumbar hernia: a case report

**DOI:** 10.1186/s13256-023-03874-5

**Published:** 2023-04-06

**Authors:** Matthieu Sumai Aza, Joël Bosomi Yawi, Jackson Kambale Musumba, Faida Kavugho Muliwavyo, Alpha Muhindo Kavuyiro, Alexendre Amini Mitamo, Claude Kasereka Masumboko, Severain Uwanda Akinja

**Affiliations:** 1 Department of Surgery, Catholic University of Graben, Butembo, Democratic Republic of Congo; 2 Department of Surgery, Official University of Mbuji-Mayi, Mbuji-Mayi, Democratic Republic of Congo

**Keywords:** Hernia, Lumbar, Bilateral Grynfeltt

## Abstract

**Introduction:**

Lumbar hernias are rare, with only 200–300 published cases listed in the literature. Two areas are described to have weakness points: the inferior lumbar triangle (Jean-Louis Petit triangle) and the superior lumbar triangle (Grynfeltt–Lesshaft triangle). Clinical diagnosis is confirmed by computed tomography and possibly by ultrasound or radiography. The surgeon must refine the clinical detection of this condition, as most patients do not have sufficient means to have a computed tomography scan performed, which remains the gold standard for diagnosis. Despite the different techniques recommended, the open route remains the most affordable in our environment.

**Case presentation:**

This case presents an 84-year-old black congolese patient consulted for bilateral swellings of the lumbar regions. The patient was married and in the farming profession for several years. The patient had no notion of trauma or fever and no notion of vomiting or stopping of materials and gases. The lumbar region presented with swellings that were ovoid, soft, painless, impulsive and expansive on coughing or hyperpressure, and non-pulsatile, measuring 9 × 7 cm in diameter (right) and 6 × 5 cm in diameter (left). Ultrasound performed of the upper costolumbal region revealed two lipomatous masses facing Grynfeltt’s quadrilateral with a 1.5 cm hole on either side. The diagnosis of bilateral Grynfeltt hernia was made, and herniorrhaphy was indicated.

**Conclusion:**

Grynfeltt–Lesshaft hernia is a rare surgical condition caused by congenital or acquired etiology. A lower back pain or a pain point localized on the hernia in addition to a lumbar mass that reduces when lying down suggests the diagnosis of lumbar hernia.

## Introduction

Lumbar hernias are rare surgical conditions; only 200–300 published cases are listed in literature [1]. The superior limit of the lumbar region is the twelfth rib, whereas the inferior limit is the iliac crest, the medial limit is the erector spinae muscles, and the external limit is the external oblique muscle. Two areas are described to have weakness points: the inferior lumbar triangle (Jean-Louis Petit triangle) and the superior lumbar triangle (Grynfeltt–Lesshaft triangle). Grynfeltt–Lesshaft triangle is an inverted triangle with boundaries formed superiorly by the twelfth thoracic rib, medially by the erector spinae muscle group, and laterally by the internal oblique muscle. The floor of this triangle is formed by aponeurosis of the transversalis muscle, and the roof is formed by the latissimus dorsi muscle [2]. The Grynfeltt triangle has three described areas of weakness: immediately below the rib where the transversalis fascia is not covered by the external oblique muscle, in the area of fascial penetration of the twelfth dorsal intercostal neurovascular pedicle, and between the inferior edge of the rib and the ligament of Henle [2–4]. Grynfeltt reported a case of hernia at the level of the upper lumbar triangle and distinguished it from the lower triangle in 1850 [5]. We report a case of bilateral lumbar hernia of Grynfeltt at the University Clinics of Graben.

## Case presentation

This case presents an 84-year-old black congolese patient consulted for bilateral painful swellings of the lumbar regions, who was married and in the farming profession for several years. There was no notion of vomiting or stopping of materials and gases, trauma, and fever.

### Clinical findings

On physical examination, the patient expressed discomfort through means of facial expression and presented with grade 3 malnutrition [calculated body mass index (BMI) 14.2 kg/m^2^]. He had a surgical history of right inguinoscrotal hernia repair 3 years ago.

The right lumbar region presented with swelling that was ovoid, soft, painless, impulsive and expansive on coughing or hyperpressure, and non-pulsatile, measuring 9 × 7 cm in diameter.

The left lumbar region presented with swelling that was ovoid, soft, painless, impulsive and expansive on coughing or hyperpressure, and non-pulsatile, measuring 6 × 5 cm in diameter.

### Timeline

The patient had noticed a mass in the Grynfeltt area for 3 years without any notion of pain nor vomiting in the beginning, and was first followed up in a health center where he had received medical treatment without success.

A few months later, he consulted a traditional practitioner for the same symptomatology where he benefited from scarification without amendment, which motivated his consultation in our hospital for better care.

### Diagnostic assessment

**Fig. 1:**
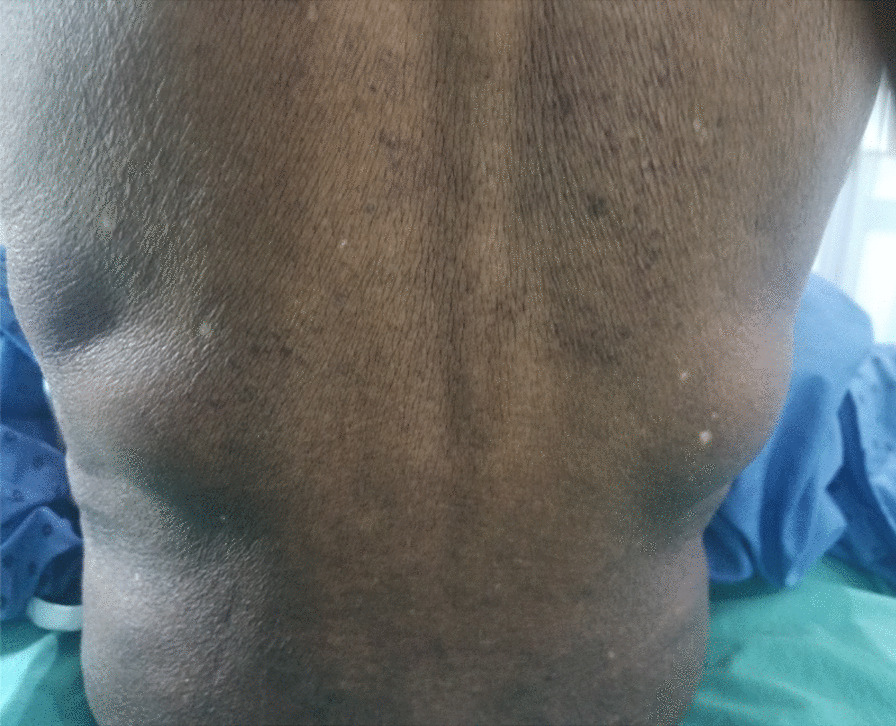
Bilateral Grynfeltt hernia

Ultrasound performed of the upper costolumbal region revealed two lipomatous masses facing Grynfeltt’s quadrilateral with a 1.5 cm hole on either side. Due to a lack of means, computed tomography (CT) scan had not been carried out. On the basis of the clinical examination and ultrasound, the diagnosis of bilateral Grynfeltt hernia was made. Bilateral herniorrhaphy was indicated (Fig. 1).

### Therapeutic intervention

**Fig. 2:**
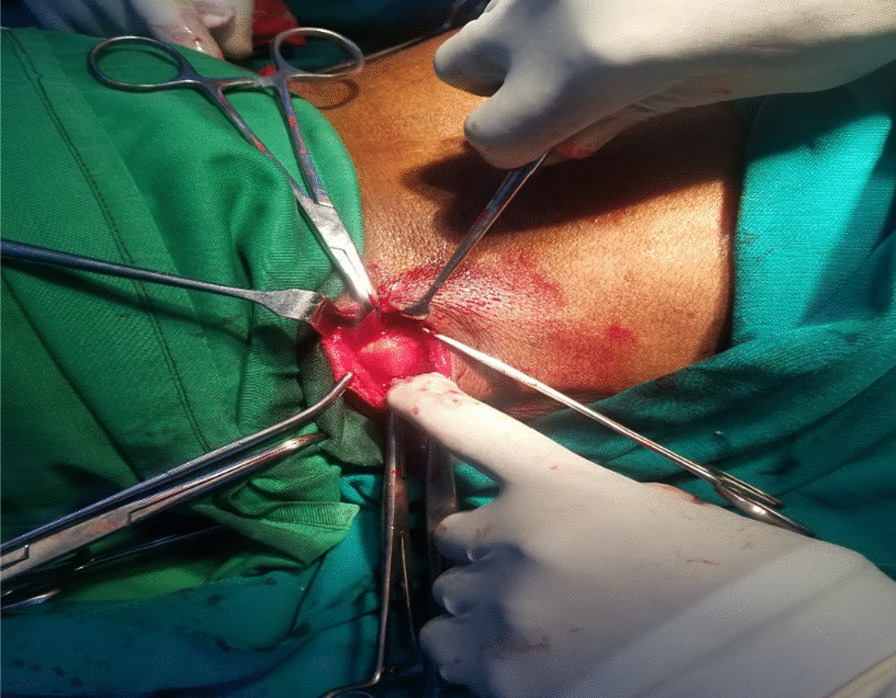
Intraoperative view demonstrating hernial sac with fatty content on the right side

**Fig. 3:**
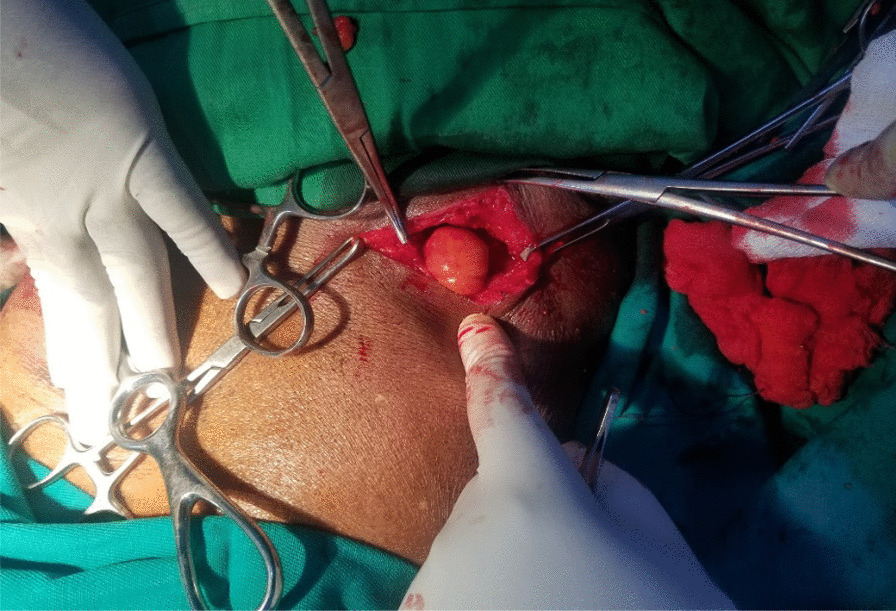
Intraoperative view demonstrating hernial sac with fatty contents on the left side

The patient was placed in prone position under general anesthesia. After two lumbar mucosal skin incisions and subcutaneous cell tissue dissection, we observed two large bilateral hernial sacs and muscle structure weakness (Figs. 2, 3).

**Fig. 4:**
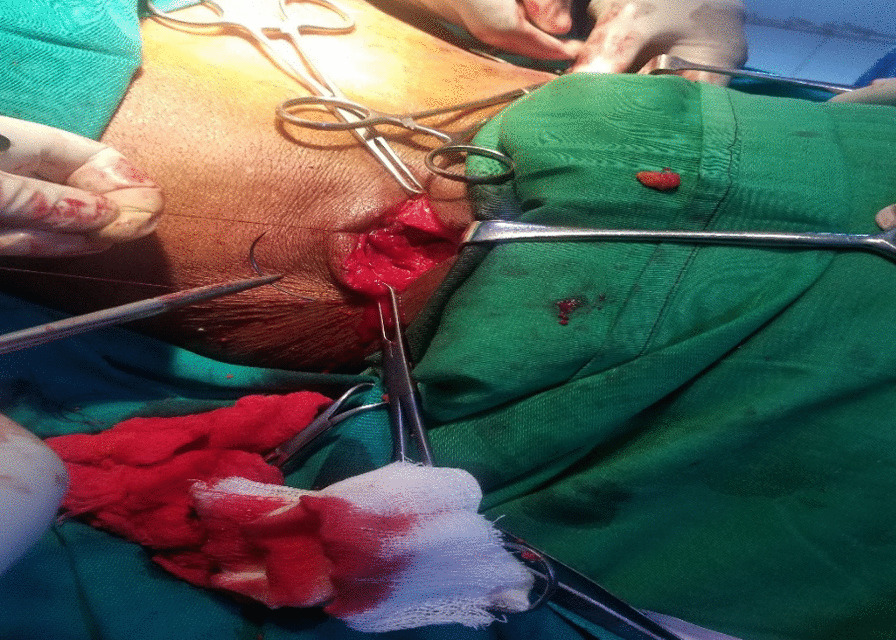
Intraoperative view. Repair of the defect with Vicryl 1

The sacs were ligated with Vicryl 2/0 and severed at their base. Both orifices were closed at surgery with resorbable Vicryl 1 suture (Fig. 4).

### Follow-up and outcomes

The patient was discharged on postoperative day 4 and the postoperative follow-up was unremarkable.

## Discussion

The diagnosis of an upper lumbar hernia is difficult because it is often confused with a lipoma or a cold abscess. This diagnostic difficulty is due to its rarity. Lumbar hernias are relatively rare [6]. The upper lumbar triangle is the weakest point of the posterior wall of the abdomen [7]. Old age and weight loss can be contributing factors [8]. Patients are usually asymptomatic. They may sometimes complain of back pain and a feeling of swelling. These swellings are initially small and gradually increase in size [9]. Congenital forms are rare (20%) while acquired lower lumbar hernias are largely secondary [10]. Several risk factors have been described for spontaneous hernias, including age, obesity, extreme thinness, intense wasting, chronic debilitating disease, muscular atrophy, chronic bronchitis, infected wound, and postoperative sepsis [11]. The age of patients is generally between 50 and 70 years [2]. Profession and thinness were found in our case. The mechanism evoked would be that the reduction in fat favors the rupture of the vasculonervous orifices that cross the dorsolumbar fascia. Situations increasing intraabdominal pressure would act as factors that trigger the appearance of these hernias [11].

Several classifications have been proposed, including that of Alfredo Moreno-Egea, which takes into account six elements (location, size, content, muscular atrophy, etiology, and recurrence) and proposes therapeutic options for each of the four types retained [12]. Clinical diagnosis of lumbar hernia requires high clinical suspicion. This depends on the size and content, which may be retroperitoneal fat, kidney, or colon and more rarely, the small intestine, omentum, spleen, ovary, or appendix [2].

When the patient consulted a local medical center 1  year ago for swelling in the lumbar region, a diagnosis of lipoma was made. This is in accordance with the literature, which supports the clinical similarity between lipoma and lumbar hernia and additionally draws the attention of the clinician to the differential diagnosis of lumbar tumefactions. This diagnosis, apart from hernia, can include lipoma, abscess, hematoma, or tumor of the soft tissues [13]. When the content is intestinal, its noise characteristic is audible on auscultation with a stethoscope. In addition, if there is strangulation, nausea, vomiting, cessation of matter and gas, and abdominal distension can be added to the symptomatology, whereas on physical examination, the swelling becomes irreducible [14].

Surgery aims to correct the defect and reconstruct an abdominal wall that is sufficiently elastic but resistant to daily physical stress. In the case of intestinal content, the risk of strangulation should always be indicated [15]. Surgical hernia repair is the ideal choice. The classic technique is as good as the laparoscopic technique, although the latter will have better results. There is not really a consensus on the best method of repairing a lumbar hernia due to its low intestinal incidence [16]. Synthetic mailplasty is the most commonly used among open repairs, combined with muscle and intestinal flaps [17].

Many operative techniques have been described, but there is no recommendation for any of these techniques or approaches. Open surgical technique has proved effective 1 year after surgery. Younger patients have increased levels of gut tissue formation [17]; but younger patients are more active, which may affect the recurrence of lumbar hernia or postoperative complications. The patient operated on followed a strict schedule in the postoperative phase that did not involve the practice of intense exercises during recovery; this fact helped reduce the likelihood of recurrence.

## Conclusion

Grynfeltt–Lesshaft hernia is a rare surgical condition caused by congenital or acquired etiology. Lower back pain or a pain point localized on the hernia in addition to a lumbar mass that reduces when lying down suggests the diagnosis of lumbar hernia. In our low-resource settings, the surgeon must refine the clinical detection of this condition, as most patients do not have sufficient means to have a CT scan performed, which remains the gold standard for diagnosis. Despite the different techniques recommended, the open route remains the most affordable in our environment.

## Data Availability

Not applicable.

## References

[1] Pélissier E, Habib E, Armstrong O. Traitement chirurgical des hernies lombaires. EMC-Techniques chirurgicales–Appareil digestif. 2010: 40–152

[2] Sharma P (2009). Lumbar hernia. Med J Armed Force India.

[3] Pachani A B, Reza A, Jadhav RV, Mathews S. A primary idiopathic superior lumbar triangle hernia with congenital right scoliosis: a rare clinical presentation and management. Int J Appl Basic Med Res. 2011;1(1). 10.4103/2229-516X.81985 PMC3657950 23776777

[4] Walgamage B, Thilan Ramesh BS, Alsawafi YB (2015). Rapport de cas et examen de la hernie lombaire. Int J Surg Case Rep..

[5] Geis WP, Hodakowski GT. Lumbar hernia. In: Nyhus L, Condon R, editors. Hernia. 5th edn. Philadelphia: 2001. p. 425–7.

[6] Mubashir AS, Aakib HC, Adil PS, Haroon RZ (2013). Lumbar hernia: an unusual presentation of bear maul. Int J Clin Med.

[7] Tchoungui Ritz FJ, Kouam V, Ticheu F (2018). Primary Jean Louis Petit and Grynfelt–Lessahaft concomitant hernias: a case report. Int J Surg Case Rep.

[8] Townsend CM, Beauchamp RD, Evers BM, Mattox KL (2017). Sabiston textbook of surgery.

[9] Ploneda-Valencia CF, Cordero-Estrada E, Castaneda-Gonzalez LG, Sainz-Escarrega VH, Varela-Munoz O, De la Cerda-Trujillo LF (2016). Grynfelt–Lesshaft hernia a case report and review of the literature. Ann Med Surg (Lond).

[10] Williams NS, Bulstrode CJK, O'Connell PR. Bailey & Love's Short Practice of surgery, 26th edn, CRC Press. 2013; 633

[11] Rajasekar M, Kumar KV. A rare case of lumbar hernia: a case report with review of literature, IOSR, e-ISSN: 2279-0853, pISSN: 2279-0861. 2015; 14(3): 10–13

[12] Moreno-Egea A, Enrique G, Miguel Calle C, Jose Antonio T, Martinez JC, Luis Albasini A (2007). Controversies in the current management of lumbar hernias. Arch Surg.

[13] Devlin B, Kingsnorth AN (1998). Management of abdominal hernias.

[14] Sahoo MR, Anil KT (2013). Sandwich technique of closure of lumbar hernia: a novel technique. Int J Case Rep Images.

[15] Meinke AK (2003). Totally extraperitoneal laparoendoscopic repair of lumbar hernia. Surg Endosc.

[16] Sundaramurthy S, Suresh HB, Anirudh AV, Prakash RA (2016). Hernie lombaire primaire: une hernie rarement rencontrée. Int J Surg Case Rep.

[17] Guo S, DiPietro LA (2009). Facteurs affectant la guérison de Woung. J Dent Res.

